# Host genotype and environmental factors differentially shape the black piranha’s gill microbiota

**DOI:** 10.1128/spectrum.03277-25

**Published:** 2026-04-30

**Authors:** Alizée Thomas, François-Étienne Sylvain, Eric Normandeau, Nicolas Leroux, Aleicia Holland, Jaqueline Costa Custódio, Pierre-Luc Mercier, Adalberto Luis Val, Nicolas Derome

**Affiliations:** 1 Institut de Biologie Intégrative et des Systèmes, Université Laval 213566 , Québec City, Québec, Canada; 2 Department of Morphology, Institute of Biological Sciences, Federal University of Minas Gerais 113014, Belo Horizonte, State of Minas Gerais, Brazil; 3 Fisheries and Oceans, Gulf Fisheries Center, Moncton, New Brunswick, Canada; 4 Plateforme de bio-informatique de l'IBIS (Institut de Biologie Intégrative et des Systèmes), Université Laval 213566 , Québec City, Québec, Canada; 5 Department of Ecology, Environment and Evolution, School of Life Science, La Trobe University, Bundoora, Victoria, Australia; 6 Laboratório de Ecofisiologia e Evolução Molecular, Instituto Nacional de Pesquisas da Amazônia (INPA) 191073, Manaus, State of Amazonas, Brazil; National Center for Genetic Engineering and Biotechnology, Khlong Luang, Pathum Thani, Thailand

**Keywords:** gills, microbiota, Amazon, genotype, fish, bacterioplankton, 16S RNA, population genetics, Genotyping-by-sequencing

## Abstract

Gills play a crucial role in teleost fish, contributing to respiration, osmoregulation, and immunity. The mucus they secrete, and their associated microbiota, support these physiological functions and serve as a first line of defense against pathogens. However, the relative influence of host genotype and environment on gill microbiota remains poorly understood, particularly under field conditions. The black piranha (*Serrasalmus rhombeus*) is a widespread Amazonian predator that harbors multiple genetic groups found in sympatry across different water types: black (acidic, tannin-rich, nutrient-poor), clear (neutral, nutrient-poor), and white (neutral, nutrient-rich, turbid). This makes it an excellent model to explore these interactions. Therefore, to assess the relative contributions of host genotype and environmental factors to gill microbiota composition, we analyzed 252 individuals sampled from 14 sites using Genotyping-by-sequencing to determine host genetic structure, and 16S rRNA gene metabarcoding to characterize the active bacterial communities in gill mucus and surrounding bacterioplankton. Our findings show that host genotype significantly affects gill microbiota composition, with distinct beta diversity and exclusive microbial taxa associated with different genetic groups, even when exposed to the same water type. Nonetheless, variation in microbiota composition was more strongly associated with site-specific environmental factors, particularly bacterioplankton communities. These results reveal a complex interplay between genetic and environmental drivers of fish-associated microbiota under natural conditions. They suggest a potentially important ecological relationship between bacterioplankton and host-associated microbiota and suggest that gill microbial communities may serve as sensitive biomarkers for detecting environmental disturbances in tropical freshwater ecosystems.

**IMPORTANCE:**

Fish gills are protected by a layer of mucus that hosts a community of beneficial bacteria essential for many vital functions. Understanding the factors that shape this gill microbiota is crucial not only for fish health but also for developing reliable microbial biomarkers to support habitat monitoring and conservation. In this study, we examined black piranhas from different genetic groups sampled across sites with contrasting water physicochemical parameters. This model provided a unique opportunity to disentangle the effects of host genetics and environmental conditions on gill microbiota composition under field conditions. Our findings show that although individuals from different genetic groups hosted distinct microbial communities, local environmental factors, especially the bacteria in the surrounding water, had a stronger influence. These results highlight the complex interplay between host and environment in shaping microbial communities and offer new insights into how fish and their microbiota respond to ecological variability in tropical freshwater ecosystems.

## INTRODUCTION

The gill is a crucial organ in the physiology of teleostean fish, fulfilling essential roles in respiration, osmoregulation, acid-base balance regulation, and the excretion of nitrate waste (1). The microbial community inhabiting the gill surface, known as the gill microbiota, appears to play a significant role in these functions. Indeed, previous studies investigating the functions of the gill microbiota have demonstrated its possible involvement in processes, such as denitrification (2), ionoregulation (3), and immunity (4–6), which is very important given that the gill serves as a primary entry point for opportunistic pathogens (7).

The composition of the gill microbiota is highly dynamic and can be influenced by multiple factors. Both abiotic and biotic elements shape the microbial communities associated with fish gills. Abiotic factors include water physicochemical parameters (3, 8–11), water microbiome (bacterioplankton) (8, 12, 13), stress (14), diet (15), as well as biotic factors, including host genotype (13, 15–17), life stage (18–20), population density (21), disease (22, 23), and parasites infection (24–26). Given their direct exposure to the aquatic environment, external body parts, such as skin and gill mucus, are likely to be particularly sensitive to the physicochemical properties of the surrounding water (8, 12, 27–29) and water microbiota (8, 13, 15, 30).

However, the extent to which the host genotype influences gill microbiota remains poorly understood. While a previous study has demonstrated significant differences in gill microbiota between species (17), within-species comparisons did not reveal a significant genotype influence on gill microbiota composition in Atlantic salmon (*Salmo salar*) (31) and rainbow trout (*Oncorhynchus mykiss*) (21). In contrast, Sylvain et al. (13) observed a significant effect of genotype in the flag cichlid (*Mesonauta festivus*), with the environment, particularly bacterioplankton, exerting a more pronounced influence.

Separating environmental influences from genetic factors in natural populations can pose significant challenges due to the potential for confounding variables. To overcome this challenge, we require a species exhibiting a wide range of genetic clusters, some of them coexisting in sympatry, and demonstrating the ability of one or a few genetic clusters to thrive in contrasting environments. The black piranha (*Serrasalmus rhombeus*) encompasses all these characteristics, making it an ideal candidate.

Indeed, it thrives in the contrasting water types found within the Amazon region, i.e., in black water (high concentration of dissolved organic carbon, pH 3.5–6, low in nutrients, low conductivity 5–20 μS cm^−1^), clear water (limpid, pH 5.5–8, and low conductivity 5–40 μS cm^−1^), and white water (pH 6.5–7, higher conductivity 40–300 μS cm^−1^, nutrient-rich, and turbid) (32–37), showcasing its adaptability to various environmental conditions. In addition, a study from Thomas et al. (38) showed that the *S. rhombeus* population in central Amazonia harbors a strong genetic structuration, with 12 putative genetic clusters distributed across 14 sites. Interestingly, numerous groups were found in sympatry, and some groups were found in sites with contrasting physicochemical parameters (black and white water). These diverse characteristics make *S. rhombeus* a particularly relevant model for studying the relative contribution of the environment to the microbiome in natural conditions.

Given the important role of gill microbiota in fish health and ecology and its potential utility as a biomarker for detecting environmental changes and disturbances, enhancing our understanding of the factors influencing the gill microbiota composition is essential (13, 30). Furthermore, in their study, Thomas et al. (38) detected an ambiguous signal suggesting the potential presence of cryptic species in *S. rhombeus* populations. However, this inference relied only on genomic data, and further evidence is needed to confirm its presence (39). Therefore, this study presents an opportunity to explore the utility of microbiota analysis in identifying potential cryptic species, an approach that has been underexplored until now. Sylvain et al. (40) demonstrated species-specific differences in gut and skin microbiota across different piranha species, suggesting that microbiota analysis could provide a distinct signal indicative of species divergence. Understanding the impact of genotype on microbiota composition offers promising insights into the potential use of microbiota analysis alongside other tools for detecting cryptic species.

Thus, our objective was to measure the relative influence of the host (genotype) and the environment (water physicochemical parameters and bacterioplankton) on *S. rhombeus* gill microbiota. We also determined if the cryptic species candidate group identified in Thomas et al. (38) showed a distinctive signature in terms of active bacterial microbiota composition. To do so, we used a metabarcoding approach based on 16S rRNA transcripts from gill microbiota and bacterioplankton. For the host genotype, we used 29,587 SNPs across the *S. rhombeus* genome from a Genotyping-by-sequencing approach (38). For the water physiochemistry, we used 34 environmental factors (3). We tested the hypothesis that host genotype plays an important role in shaping the gill microbiota, while environmental factors, particularly bacterioplankton, have a stronger impact (13). In addition, we expected individuals suspected of belonging to a potential cryptic species (38) to show a distinctive signal in terms of active bacterial microbiota composition.

## MATERIALS AND METHODS

### Sampling

**Fig 1:**
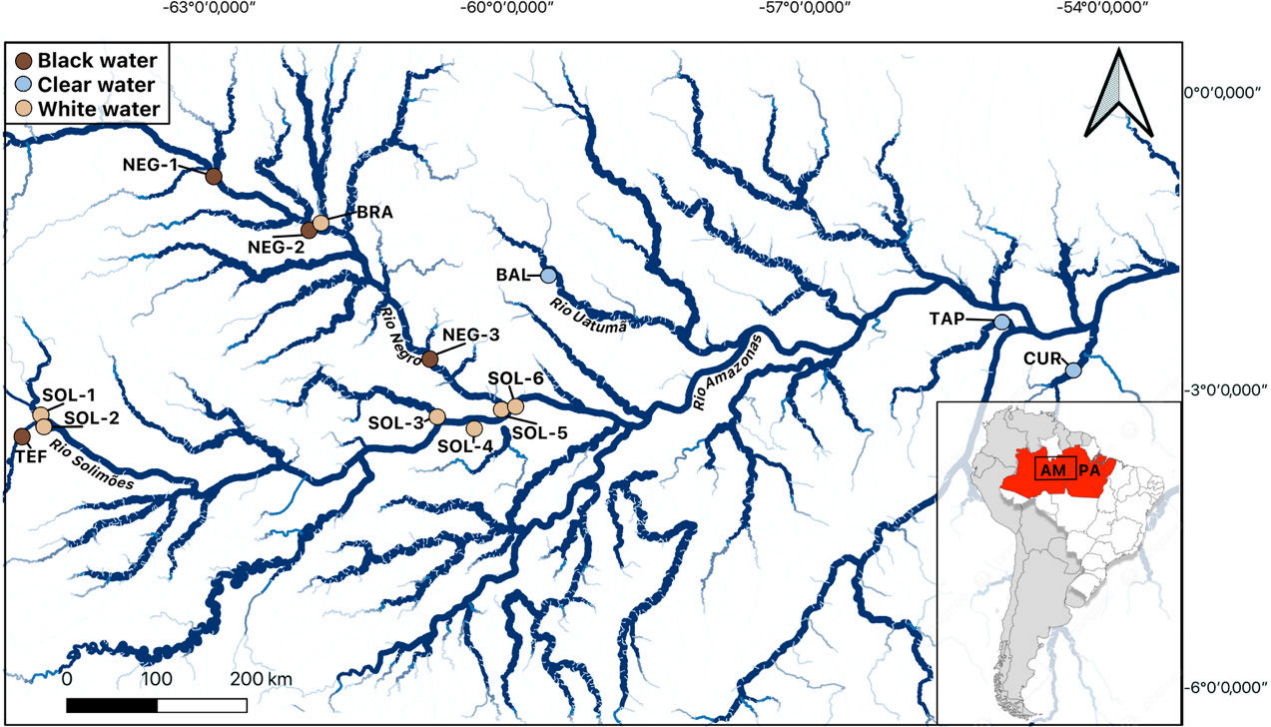
Map of sampling carried out in 2018 and 2019 in the central Amazon, in the state of Amazonas (AM) and Pará (PA), Brazil. The map was made with QGIS software version 3.24.3 with the GPS coordinates of the sampling sites and hydrographic data from the *catálogo de metadados da Agência Nacional de Águas—Instituto Brasileiro de Geografia e Estatística*.

A total of 252 individuals of *S. rhombeus* were collected using line fishing and gillnets in the central Amazon region, within the states of Amazonas and Pará in Brazil in 2018 and 2019 during the dry season at 14 sites (Table S1) on different tributaries of the Amazon (3), including seven whitewater, four blackwater, and three clearwater sites (Fig. 1). Fin and gill clip samples were collected and preserved in nucleic acid preservation buffer to preserve maximum DNA and RNA integrity at room temperature (41). All collected specimens were in apparent good health. The samples were then stored at −20°C. Immediately after conducting fish sampling, a sample of environmental water was collected, and 34 physicochemical parameters were measured (Tables S3 to S5) (detailed methodology in reference 3). Water types (clear, white, and black water) were determined by combining physicochemical measurements (Tables S3 to S5) with established hydrochemical definitions of Amazonian water types, as described in the literature (32–37). The fieldwork was done under the permits SISBIO 29837-12 and CPAUL 2018021-1. Sample exportation was done under the permit 00000481/2028-UVGAMAO-AM.

### DNA and RNA extraction

We first dissected the fin tissue (20 mg) and placed the pieces in a 10× PBS solution and incubated them in a thermocycler at 56°C for a minimum of 8 h. We then performed a DNA extraction from the fin tissue using the *QIAGEN* DNeasy Blood and Tissue kit, according to the manufacturer’s instructions, without modification, at the *Instituto Nacional de Pesquisa da Amazonia* (INPA) (see reference 38 for methodological details). The DNA solutions were preserved at −20°C. To obtain the water communities, 2 L of water, taken 30 cm from the surface, were sampled at each site in sterile Nalgene bottles and then filtered through 0.22 µm-pore size polyethersulfone Sterivex filters (cat #SVGP01050, Millipore, Burlington, MA, USA), following the method of Cruaud et al. (42). RNA was extracted from the gill tissues and communities present in the water using the TRIzol protocol, without modification, at the Institut de Biologie Intégrative et des Systèmes (IBIS), Université Laval. The samples were then stored at −80°C. Reverse transcriptase was then performed to obtain DNA complementary to the RNA fragments. The RNA data used in this study corresponds to those previously published in Sylvain et al. (3), to which we refer for a detailed description of the methodology.

### Genotyping-by-sequencing

To provide genotypic information from the fish, Genotyping-by-sequencing library preparation was done following the protocol by Abed et al. (43). Briefly, DNA from fin samples underwent digestion with *PstI* and *MstI* restriction enzymes in Cutsmart buffer, followed by ligation using T4 DNA Ligase reaction buffer. After purification with the QIAquick PCR Purification kit, DNA fragments were amplified with a PCR (see reference 43 for the primers used). The amplified fragments were purified using Axygen magnetic beads and assessed for quality with a BioAnalyzer. Sequencing was performed using the *Ion Proton sequencer* at the Plateforme d’Analyses Génomiques, Integrative and Systems Biology Institute (IBIS), Laval University. SNP calling was performed *de novo* with STACKS v2.62. See Thomas et al. (38) for methodological details.

### Metabarcoding

The 16S rRNA gene transcript libraries were prepared through PCRs, with amplification of the V3–V4 region, using the forward and reverse primers 347 F (5′-GGAGGCAGCAGTRRGGAAT-3′) and 803 R (5′-CTACCRGGGTATCTAATCC-3′) (44). The use of 16S rRNA transcripts abundance allows estimation of relative microbial activity within the detected taxa, as rRNA abundance reflects transcriptional activity (45). A first PCR was conducted, with the following conditions: an initial denaturation step of 2 min at 98°C, followed by 30 cycles of 10 s at 98°C, 30 s at the optimal temperature (60°C), 30 s at 72°C, and a final elongation step of 2 min at 72°C. Then, after purification using Axygen magnetic beads (Beckman Coulter Genomics), a second PCR was performed to add molecular barcode labels. The second PCR amplicons were done in similar conditions, with an initial denaturation step, followed by nine cycles of amplification. Negative controls were utilized for reverse transcription and PCR to ensure no contamination during sample handling. Subsequently, the individually amplified and labeled products from the second PCR were pooled in equal proportions and submitted to the Genomic Analysis Platform at the Institute of Integrative and Systems Biology (IBIS), Laval University, for sequencing on an Illumina MiSeq platform (2 × 300 bp paired-end reads). Regarding the bioinformatics analyses, the raw sequences were profiled according to quality per nucleotide using the fastQC program. The sequences were then analyzed with the R DADA2 package to check for quality, detect sequencing errors, infer amplicon sequence variants (ASVs), and identify chimeras. Taxonomic assignment was carried out by sequence alignment using BLASTn based on the NCBI database for 16S regions. The 16S sequencing data used in this study were previously published, with full methodological details provided in Sylvain et al. (3).

### Statistical analysis

To test the relative effect of host (genotype) and the environment (site and water type [black, white, clear water]) on active gill microbiota composition, beta diversity analyses were carried out to examine the level of taxonomic differentiation between individuals in genetic groups using Bray-Curtis distance. We visualized the distance matrices between groups using principal coordinates analysis (PCoA) and tested the difference in beta diversity between groups using PERMANOVA, with the R package *vegan*. For each test conducted, we also carried out a *Betadisper* test. This allowed us to assess whether the points of different groups were dispersed differently around their respective centroids, potentially affecting the significance of the PERMANOVA analysis. This test was also performed with the R package *vegan*. We conducted tests within each water type, using both site and genotype as explanatory variables. To mitigate potential confounding effects with the site, tests were also conducted within sites containing multiple genetic clusters and with a minimum of three samples from each cluster. This approach enabled us to isolate the genotype effect from site factors. Additionally, we performed tests on genetic clusters present at multiple sites to assess their respective environmental contribution in shaping active gill microbiota composition.

We used the *multipatt* function from the indicspecies R package to identify ASVs that were significantly associated with specific genetic groups. We also performed a Mantel test to study the correlation between the gill microbiota transcriptional activity (Bray-Curtis distances) and (i) bacterioplankton transcriptional activity (Bray-Curtis distances), (ii) host genetic distances (1 − F_ST_/F_ST_), and (iii) environmental parameters (Euclidean distance) on the microbiota composition. In addition, we performed a Mantel test between bacterioplankton transcriptional activity (Bray-Curtis distances) and environmental parameters (Euclidean distance). For the environmental parameters, we used parameters that were not highly correlated with each other. We then normalized and centralized the variables and calculated Euclidean distances between each pair of sites. The Mantel analysis was performed with the R package *Vegan* with 1,000 interactions. We also performed the correlation between gill microbiota transcriptional activity and host genetics (utilizing genetic distance calculations following the method by Kosman and Leonhard [46]) at each site that contained several genetic clusters.

In addition, we conducted a linear mixed effects regression (LMER) to study simultaneously the effect of different explanatory variables. The explanatory variables, used as fixed effects, were the same as the ones used for the Mantel test (host genetic, bacterioplankton, physicochemical parameters). The response variable was the gill microbiota transcriptional activity (Bray-Curtis distances). We performed different models, using all possible combinations with one, two, and three explanatory variables, and their possible interactions. We also performed models considering the interaction between bacterioplankton and physicochemical parameters, in addition to the direct effect on active gill microbiota composition. A null model, without the fixed effects, was also conducted. The LMER was performed with the R package *lme4*. We selected the models that had the highest AIC weight values relative to the null model, calculated with the *AICcmodavg* R package. We then used the R package *indicspecies* to detect ASVs that were exclusive to specific genetic groups. We performed it for groups that were in sympatry at the same site, to avoid a confounding effect with the environmental factors. Finally, we conducted interaction network analyses between gill microbiota ASVs and physicochemical parameters with the following similarity measures: Bray and Curtis, Kullback–Leibler dissimilarity measures, Pearson and Spearman correlation, and mutual information (47, 48), using the CoNet plugin in the Cytoscape software. We used a correlation threshold of 0.5, 1,000 iterations, and Bonferroni corrections. Such analyses allow the detection of a correlation between each ASV and physicochemical parameters.

## RESULTS

### Microbiota composition

**Fig 2:**
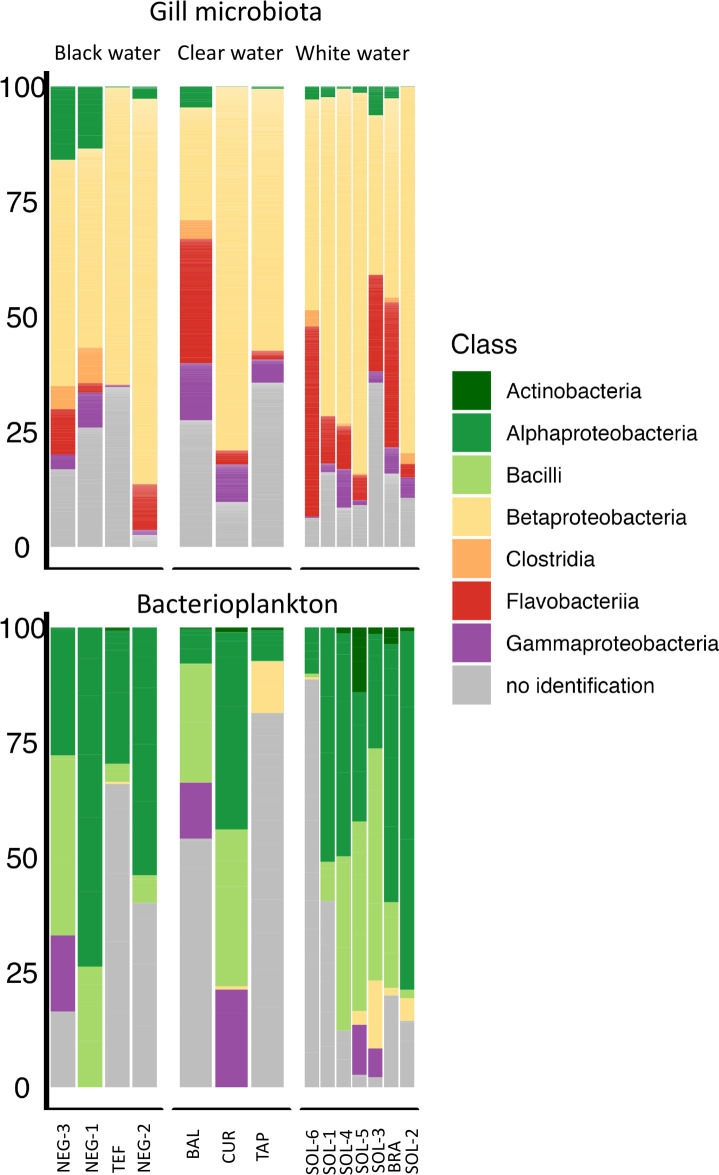
16S rRNA transcripts relative abundance of the 100 most active ASV at the Class level in *Serrasalmus rhombeus* gill microbiota and water communities (bacterioplankton). Samples are clustered according to their sampling site of origin.

The most active bacterial classes from the gill microbiota of black piranha were *Betaproteobacter*ia (59.18% [including 9.62% of *Burkholderiales* and 4.98% of *Neisseriales*], *Flavobacteriia* 12.53% [all *Flavobacteriales*], and 4.44% *Gammaproteobacteria* [1.31% *Enterobacterales*, 2.64% *Pseudomonadales*]) (Fig. 2; Fig. S1). Among the 100 most active ASVs based on 16S rRNA transcripts relative abundance, a mean of 90.57% was unidentified at the species level, 89.16% at the genus level, 72.55% at the family level, 65.48% at the order level, 18.26% at the class level, 13.10% at the phylum level. The most active classes within the bacterioplankton were *Alphaproteobacteria* (19.51% *Rhizobiales*, 2.61% *Sphingomonadale*), *Bacilli* (12.67% *Baccillales*, 8.39% *Lactobacillales*), and *Gammaproteobacteria* (4.79% *Pseudomonadales*) (Fig. 2; Fig. S1).

### Relative effect of environment and genotype

**Fig 3:**
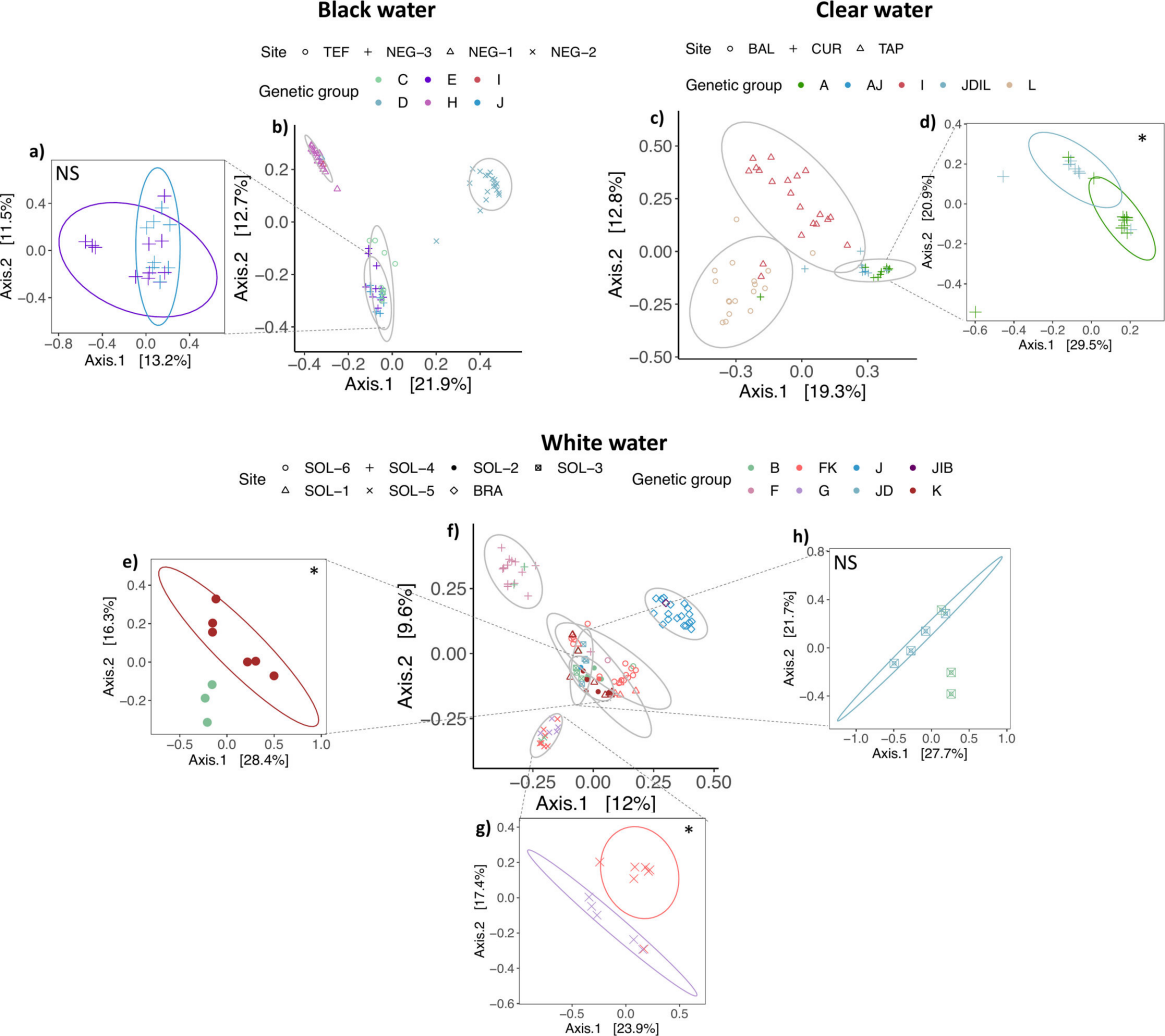
PCoA on *Serrasalmus rhombeus* gill microbiota communities according to Bray-Curtis distance for (**a**) genetic groups E and J within the site NEG-3, (**b**) the sites found in black water, (**c**) the sites found in clear water, (**d**) the genetic groups JDIL and A in the site CUR, (**e**) the genetic groups B and K in the site SOL-2, (**f**) sites in white water, (**g**) the genetic groups FK and G in the site the site SOL-5, and (**h**) the genetic groups JD and B in the site SOL-3. In the figures **b**, **c**, and **f**, the ellipses were calculated according to the site; meanwhile, in the figures **a**, **d**, **e**, **h**, and **g**, the ellipses were calculated according to the genotype groups. The asterisks correspond to the PERMANOVA performed, where NS = *P* value > 0.05 and * = *P* value < 0.05.

The PERMANOVA performed on the dissimilarity matrix according to the site (Fig. 3b, c, and f) was significant (in black water: R^2^ = 0.37, F_3,63_ = 12.18, *P* = 0.001, in clear water: R^2^ = 0.26, F_2,64_ = 9.34, *P* = 0.001, in white water: R^2^ = 0.37, F_7,92_ = 9.59, *P* = 0.001). The PERMANOVA performed on the dissimilarity matrix according to the genotype was also significant (in black water: R^2^ = 0.40, F_5,61_ = 6.64, *P* = 0.001, in clear water: R^2^ = 0.28, F_4,51_ = 4.98, *P* = 0.00, in white water: R_2_ = 0.26, F_6,10_ = 4.67, *P* = 0.001). The PERMANOVA performed according to the genetic groups within the same site (Fig. 3a, d, e, g, and h) was significant for groups in site CUR (R^2^ = 0.11, F_1,16_ = 1.98, *P* = 0.02, Fig. 3d), SOL-2 (R^2^ = 0.19, F_1,7_ = 1.42, *P* = 0.17, Fig. 3e) and SOL-5 (R^2^ = 0.16, F_1,10_ = 1.66, *P* = 0.001, Fig. 3g). On the other hand, the genetic groups within the site NEG-3 and SOL-3 did not show a significant difference in gill microbiota composition (R^2^ = 0.06, F_1,17_ = 1.07, *P* = 0.36 for NEG-3 and R^2^ = 0.21, F_1,5_ = 1.32, *P* = 0. 097 for SOL-3).

**Fig 4:**
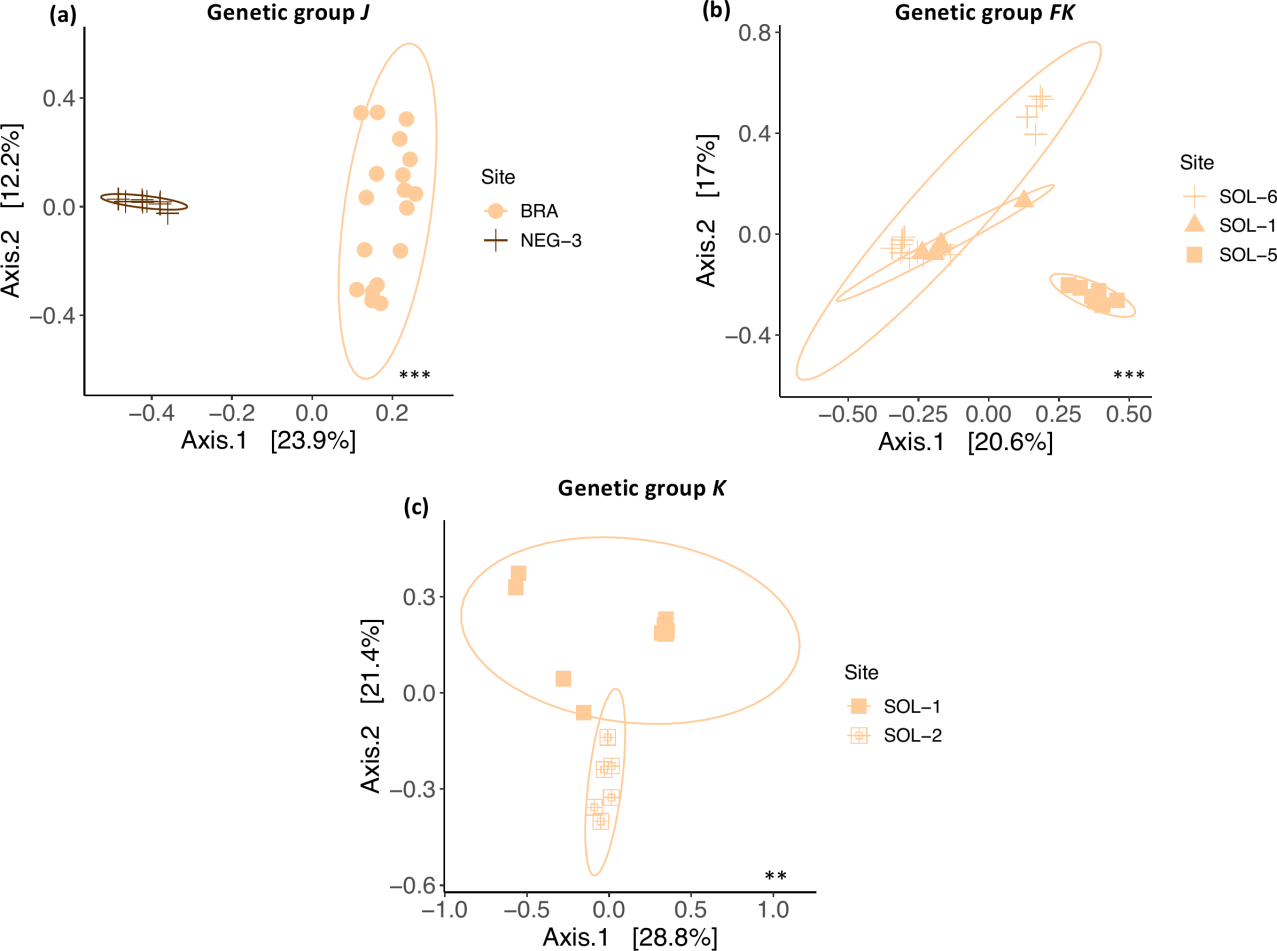
PCoA based on Bray-Curtis distances of gill microbiota from individuals belonging to genetic groups (**a**) J, (**b**) FK, and (**c**) K inhabiting two to three different sites. The asterisks correspond to the PERMANOVA results, where ** means a *P* value < 0.010 and *** means a *P* value < 0.001.

While group J exhibited no significant variance compared to group E, both found at the same site (NEG-3) (Fig. 3a), there was a significant difference between individuals from group J located at different sites (R^2^ = 0.23, F_1,24_ = 7.33, *P* = 0.001, Fig. 4a). Also, for the groups K and FK, individuals showed different microbiota composition when encountered at the same water type but in different sites (Fig. 4b and c) (K: R^2^ = 0.19, F_1,13_ = 3.008, *P* = 0.007, FK: R^2^ = 0.27, F_2,25_ = 4.80, *P* = 0.001, Fig. 4b). The *betadisper* performed showed a significant difference in the point distribution around their respective centroids for the J group (Fig. 4a) located at different sites (R^2^ = 0.70, F_1,24_ = 55.72, *P* = 0.001) (Fig. S3a). The FK group (Fig. 4b) also showed a significant difference in dispersion for the individuals at the site SOL-6 (R^2^ = 0.46, F_1,24_ = 10.68, *P* = 0.002) (Fig. S3b).

### Multipatt analysis

The *multipatt* analysis detected specific ASVs for different genetic groups living in sympatry. Overall, for the SOL-5 site, 33 ASVs were specific to the G group: one *Pelomonas saccharophila,* three *Flavobacterium*, five *Burkholderiales*, 16 *Betaproteobacteria*, two *Bacteroidetes,* and an unannotated ASV. At the same site, group FK had five specific ASVs, including two *Betaproteobacteria*, two *Bacteroidetes,* and one unannotated ASV. For the site NEG-3, group E had seven specific ASVs, including one *Aquitalea magnusonii,* two *Betaproteobacteria,* and four *Proteobacteria*. Group J had six specific ASVs, including one *Clostridiales*, one *Bacteroidetes*, three *Betaproteobacteria,* and one unannotated bacterium. At the CUR site, group JDIL contained 62 exclusive ASVs, including one *Massilia consociata,* one *Moraxella osloensis*, one *Rivicola pingtungensis,* one *Flavobacterium*, three *Moraxellaceae*, one *Bacillales*, two *Bacteroidia*, 29 *Betaproteobacteria*, one *Clostridiales*, one *Enterobacterales*, eight *Proteobacteria,* and 12 unannotated bacteria. Group A had 108 exclusive ASVs, including 102 *Betaproteobacteria* and six *Proteobacteria*. In the SOL-2 site, group F had two specific ASVs, corresponding to two *Proteobacteria*. Group B at the site SOL-2 had 12 exclusive ASVs, composed of one *Acinetobacter bereziniae,* one *Acinetobacter*, one *Bacteroidetes*, seven *Proteobacteria,* and three unannotated bacteria. At the SOL-3, group B had nine exclusive ASVs, including seven *Burkholderiales*, one *Betaproteobacteria,* and one unannotated bacteria. The group JD had only one exclusive ASV, a *Cytophagales*.

### Mantel tests

**Fig 5:**
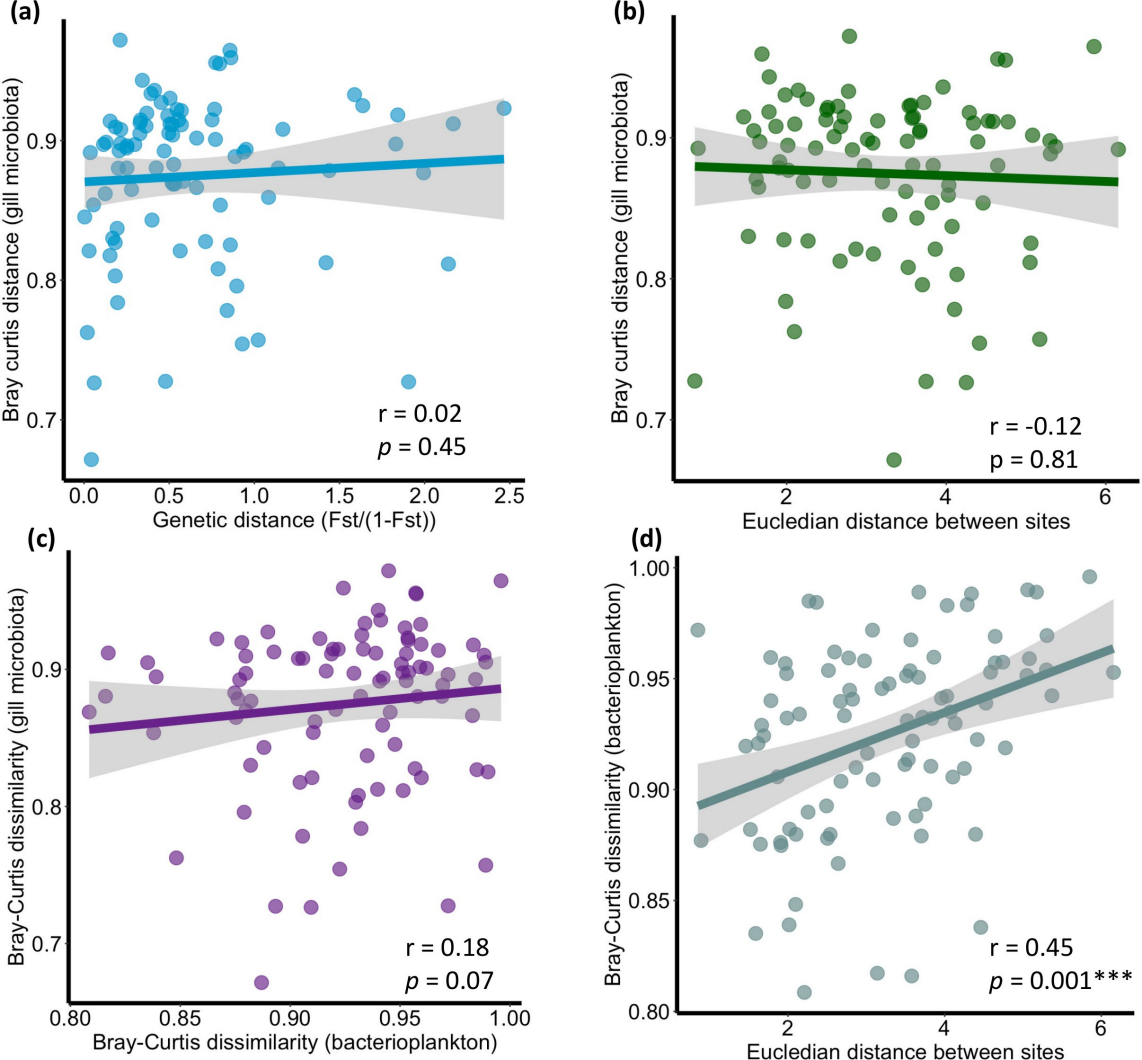
Mantel test between Bray-Curtis dissimilarity of *S. rhombeus*’ gill microbiota 16S rRNA transcripts relative abundance between sites and (**a**) the genetic distance between sites, (**b**) the Euclidean distance of physicochemical parameters between sites, (**c**) Bray-Curtis dissimilarity of bacterioplankton 16S rRNA transcripts relative abundance between sites. (**d**) Mantel test between Bray-Curtis dissimilarity of bacterioplankton 16S rRNA transcripts relative abundance between sites and the Euclidean distance of physicochemical parameters between sites.

Mantel tests were not significant for the relationship between gill microbiota activity (Bray-Curtis distance) and genetic distance (1 − F_ST_/F_ST_), nor with physicochemical parameters differences between sites (Euclidean distance). However, there was a tendency for the relationship between gill microbiota activity (Bray-Curtis distance) and bacterioplankton activity (Bray-Curtis distance) composition (r = 0.18, *P* = 0.07). The only significant Mantel test was for the relationship between bacterioplankton activity and physicochemical parameters differences between sites (r = 0.45, *P* = 0.001***) (Fig. 5). The results for the gill microbiota activity and the genetic distance between individuals at the same site were not significant for SOL-3, SOL-2, NEG-3, CUR, and SOL-5. Among the LMER tests, the only one that had an AIC weight value greater than the null model (AIC = 0.33, model null AIC = 0.17) contained the following explanatory variables: bacterioplankton and physicochemical parameters and interaction between bacterioplankton and environmental distances (Table S6). See Fig. S4 for the predicted dissimilarity values by the global model (including all variables).

### Interaction networks

**Fig 6:**
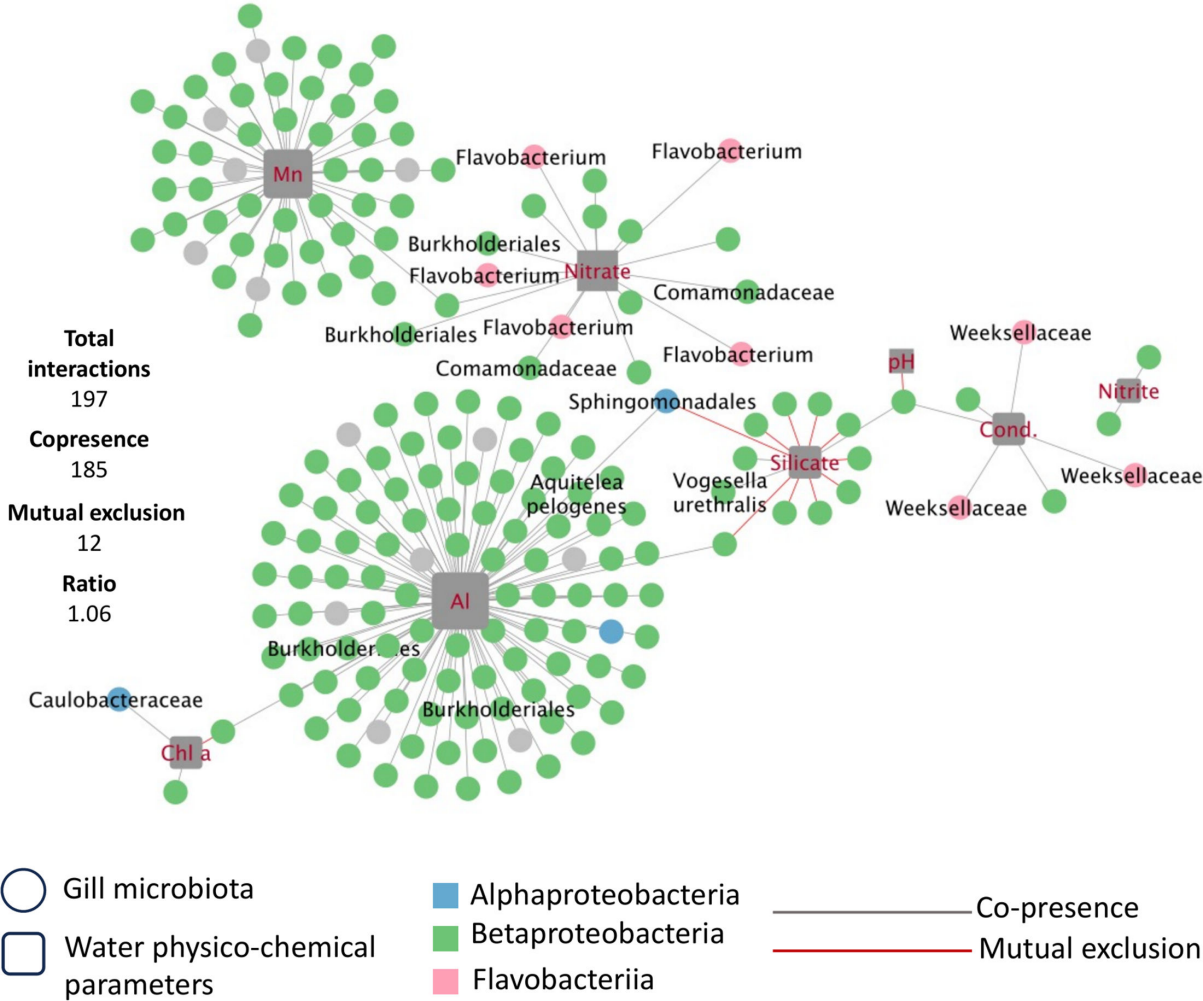
Interaction network between bacteria 16S rRNA transcripts relative abundance from gill microbiota and physicochemical parameters. Each edge between an ASV (round shape) and a physicochemical parameter (square shape) means a significant interaction. The taxa names correspond to the highest level possible for each node; a node with no name signifies that the highest taxa possible was the Class. The shape size is proportional to the number of interactions of this node with other nodes. Gray lines indicate co-activity (positive relationships), and red lines indicate mutual inhibition (negative relationships).

The interaction network (Fig. 6) shows that aluminum (Al), manganese (Mn), nitrate, silicate, productivity (Chl-a), pH, conductivity (cond.), and nitrite are significantly correlated with ASV’s activities from the gill microbiota. The aluminum had the most positive interactions, with a total of 104 positive interactions with ASVs from *S. rhombeus*’ gill microbiota, including 95 with Betaproteobacteria (two *Burkholderiales* and one *Aquitalea pelogenes)*, two with *Alphaproteobacteria* (including *Sphingomonadales*)*,* and nine unannotated ASVs. The manganese had a total of 46 positive interactions, 40 with *Betaproteobacteria* and six with unannotated ASVs. The nitrate harbored 17 positive interactions, including 12 with *Betaproteobacteria* (two *Comamonadaceae* and two *Burkholderiales*) and five interactions with *Flavobacteriia* (*Flavobacterium*). The silicate had nine negative interactions, including one with an *Alphaproteobacteria* (*Sphingomonadales*, the same ASV previously positively impacted by Al) and eight with *Betaproteobacteria*. Silicate also showed positive interaction with three *Betaproteobacteria*, including one *Vogesella urethralis*. Conductivity showed three positive interactions with *Weeksellaceae* and three with *Betaproteobacteria*. Nitrite had two positive interactions with *Betaproteobacteria,* and pH had one positive interaction with one *Betaproteobacteria*. Finally, chlorophyll a positively interacted with two *Betaproteobacteria* and one *Alphaproteobacteria* (*Caulobacteraceae*).

## DISCUSSION

This study aimed to determine the relative effect of environmental factors (physicochemical parameters and bacterioplankton) and host genotype on gill microbiota activity. Understanding these influences is essential, not only because of their fundamental role in fish health, but also to develop reliable microbial biomarkers for fish habitat conservation. The presence of multiple genetic clusters of *Serrasalmus rhombeus* living in sympatry within the same environment, as well as across environments with contrasting water types, provided a unique opportunity to disentangle the effects of genotype and environment on gill microbiota in field conditions. The PERMANOVA results showed a significant effect of the genotype, while the F and R^2^ values were higher for the environmental factors (site). *Multipatt* analysis revealed exclusive ASVs for each genetics group found under the same site factors. The Mantel and LMER tests were not significant for the genotype effect and suggested a combined effect of physiochemical parameters and bacterioplankton. The gill microbiota, particularly the class *Betaproteobacteria*, showed significant correlations with physicochemical parameters, such as aluminum, manganese, nitrate, and silicate.

### Microbiota characterization and putative functions

The black piranha’s active gill microbiota was characterized by a 16S rRNA transcripts relative abundance of *Alphaproteobacteria* (*Sphingomonadales*, *Caulobacterales*), *Betaproteobacteria* (*Burkholderiales*, *Neisseriales*), *Gammaproteobacteria* (*Enterobacterales*, *Pseudomonadales*), *Flavobacteriia* (*Flavobacteriales*) and *Clostridia* (Fig. 2). A previous study conducted on other Amazonian fishes’ gill microbiota, including the flag cichlid (*Mesonauta festivus)*, freshwater sardines (*Triportheus albus*), and peacock bass (*Cichla* spp.), showed high activity of *Proteobacteria* (*Betaproteobacteria*, *Alphaproteobacteria*, *Gammaproteobacteria*), *Actinobacteria*, and *Bacteroidetes* (*Flavobacteriales*), *Firmicutes* (*Clostridia*) (3, 13). These taxa seem to be characteristic of teleostean fish gills, as other studies on gill microbiota from different biomes also showed a predominance of those bacteria (17, 22, 49–51). In studies conducted on saltwater and freshwater species (reviewed by Sehnal et al. [30]), *Burkholderiales, Enterobacteriales, Flavobacterales, Pseudomonadales,* and *Sphingomonadales* have been shown to play a role in immunity and stress response. In addition, a study from Sylvain et al. (3) showed that *Vogesella* and *Aquitalea* (*Betaproteobacteria*) could help to maintain gill permeability in Amazonian black waters. Indeed, Sylvain et al. (3) demonstrated that rRNA transcripts from the betaproteobacterial genus *Aquitalea* were differentially abundant in blackwater. *Aquitalea* has been linked to host–microbe interactions through the production of indole, which could possibly influence ion channel activity and enhance epithelial tight-junction resistance (52–54). In addition, in *S. rhombeus*, expression of an ATP-sensitive K^+^ transporter was correlated with porB expression by *Aquitalea* and *Vogesella*, a major outer membrane porin known to interfere with host membrane ion selectivity.

However, our data contained a high amount of unannotated ASVs, therefore limiting our ability to infer the ecological significance of microbiota differences. Despite the advantages of metabarcoding, its limitation in taxonomic identification underscores the need for further investigation on the detected taxa and their functions. Metagenomic and metatranscriptomic approaches could offer deeper insights into species-specific functions and interactions within Amazonian fish microbiota.

### Genotype effect

Our analysis showed an effect of genotype on active gill microbiota for individuals present at the same site, which were thus exposed to the same parameters. Indeed, our PERMANOVA test was significant for the sites CUR, SOL-2, and SOL-5. However, it was not significant for NEG-3 and SOL-3 sites. For the SOL-3 site, a trend was observed, and the lack of statistical significance could be attributed to the limited sample size. Regarding the NEG-3 site, we hypothesized that environmental selection may be more important in black water, considering its harsher environment. Thus, the genotype effect would be less important in black water than in white water. However, since we only had one blackwater site with different genetic groups, further studies would be needed to validate this hypothesis.

This pattern is particularly striking in the case of NEG-3 (genetic groups J and E) and TEF (genetic group C), which are separated by over 800 km (Fig. 3a) and belong to genetically distant groups (38). The observed similarity in bacterial composition likely results from exposure to the same water type, regardless of host genetic background, highlighting the key role of the environment in structuring host-associated bacterial microbiota and providing an example of adaptive convergence among bacterial symbionts.

The Mantel test showed no significant relation between genetic distance and gill microbiota activity when using the distances between sites and between individuals from the same sites. This result may stem from the fact that these analyses assess overall genetic distance and Bray-Curtis dissimilarity. If certain taxa are impacted by distinct genetic variations, the signal may be obscured in such a test. Indeed, the *multipatt* analysis revealed that when the individuals are under the same site-factor effect, some ASVs are specific to certain genetic groups. However, due to limited resolution, we could not identify numerous ASVs beyond the order or class level, thereby limiting the depth of our interpretation.

Nevertheless, while our results suggest a significant effect of the genotype, PERMANOVA analyses suggested a higher role of site-specific factors. Indeed, the difference between individuals from the group J found at two different sites and water types was much higher (Fig. 4a) than the difference between the group J and H within the same site. The same tendency was observed for the groups K (Fig. 4c) and FK (Fig. 4b), also present at different sites but from the same water type, showing the importance of site-related factors. However, the *Betadisper* test was significant for the J genetic group and FK, indicating a different point dispersion around their centroid. Such a difference could potentially influence the PERMANOVA result, leading to a type I error. Yet, the distinct clustering observed in the PCoA plots (Fig. 4a and b) underscores that, despite variations in dispersion, the PERMANOVA results seem to reflect the differences in community composition between the groups.

Hence, our findings indicate a greater influence of site-specific factors on gill bacterial microbiota activity compared to host genotype, which is consistent with previous research findings. Indeed, while host genotype had a significant effect on gill microbiota activity in *Mesonauta festivus*, environmental factors exerted a stronger influence (13). Similarly, host genotype did not significantly affect gill microbiota composition in Atlantic salmon (*Salmo salar*) when comparing two strains reared under different hatchery systems (31), nor in rainbow trout (*Oncorhynchus mykiss*) when comparing resistant and susceptible genetic lines (21). Also, Sylvain et al. (29) demonstrated that in the flag cichlid (*M. festivus*), the pacu (*Mylossoma duriventre*), and the black piranha (*S. rhombeus)* species, skin mucus microbiota was largely shaped by environmental physicochemical conditions and bacterioplankton composition, whereas gut microbiota was more strongly influenced by host species factor. Consistently, a parallel study conducted on the Amazonian fish *M. festivus* reported a highly significant genotype effect on gut microbiota composition, which was approximately twice as strong as the environmental effect (24). Taken together, these findings suggest that host genotype exerts a stronger influence on internal microbial communities, such as those in the gut, while external microbiota associated with gills and skin are more strongly shaped by environmental factors (15, 24, 55).

### Site-specific effect

In our case, while the Mantel test did not show any significant correlation between physicochemical parameters and gill microbiota activity (Fig. 5b), the interaction network showed significant co-variation between physicochemical parameters and 197 ASVs (Fig. 6). Gill-associated bacteria activity was mostly influenced, in decreasing order, by aluminum, manganese, nitrate, and silicate. More precisely, aluminum correlated most strongly with *Betaproteobacteria* (specifically *Burkholderiales*). In a previous study, *Burkholderiales* have shown a high tolerance to high aluminum concentration (56). Also, Kunito et al. (57) demonstrated that Al-resistant bacterial populations increase with increasing soil Al levels, with the majority of isolates belonging to *Burkholderia*. In addition, *Burkholderiales* 16S rRNA transcripts were observed to increase in abundance in the presence of aluminum in tea plant roots (58).

Manganese was correlated with *Betaproteobacteria*, as previously documented in marine sediments (59) and water microbiota (60). Nitrate was correlated with the *Flavobacterium* genus and *Betaproteobacteria* class (*Burkholderiales* and *Comamonadaceae*). Approximately half of the species within the *Flavobacterium* genus are able to reduce nitrate to nitrite to supplement their energy metabolism (61). Additionally, Kutvonen et al. (62) have demonstrated that *Burkholderiales* are positively affected by nitrate abundance, and Long et al. (63) showed that *Comamonadacea*e are denitrifiers, thus elucidating the positive correlations between those taxa and nitrate detected in our data set. However, the silicate showed negative interactions with some *Betaproteobacteria* (Fig. 6). Contrary findings have been reported in previous studies, with some indicating an increase in *Betaproteobacteria* associated with silicate in soil (64), while others found no correlation in seawater (65). This disparity may stem from the limited taxonomic resolution available in our data, as we only have information at the class level for these interactions. Consequently, the observed trends may vary depending on the specific species or strain involved. In addition, the different context of microbial ecology in the above-mentioned studies may lead to differences in the way physicochemical parameters interact with taxa.

**Fig 7:**
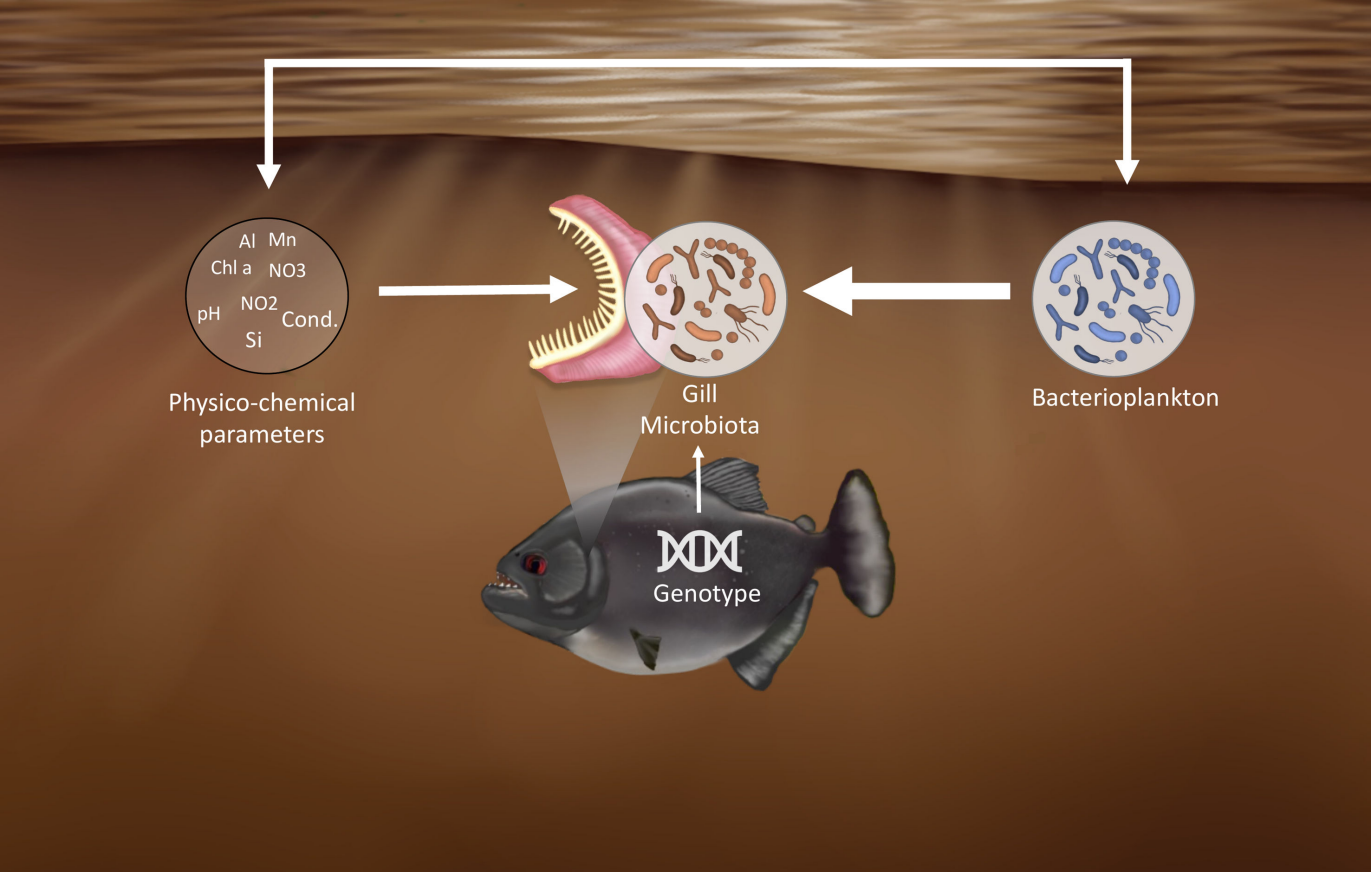
Conceptual representation of the relative influence of multiple factors shaping the gill microbiota of the black piranha (*Serrasalmus rhombeus*). Bacterioplankton appears to be the primary driver, followed by physicochemical parameters and host genotype. Moreover, the results showed an interaction between physicochemical parameters and bacterioplankton. Illustration by Alizée Thomas.

The notable disparity between sites was evident on the PCoA (Fig. 4), even for sites with close physicochemical parameters (same water type). Bacterioplankton appeared to be particularly sensitive to these parameters (see Fig. 5d). Variations in bacterioplankton composition across sites could therefore account for the differences observed in the gill microbiota between sites, possibly combined with other site-specific factors not addressed in this study. Indeed, our LMER tests indicated a direct influence of physicochemical parameters and bacterioplankton on the gill microbiota activity. Also, the physicochemical parameters and bacterioplankton would exert an indirect influence on the gill microbiota through their interactions (Table S6). Considering the large volume of water passing over fish gills, microorganisms are continuously exposed to the gill surface; however, most environmental bacteria are likely transient, while the persistent gill-associated microbiota is shaped by long-term host–microbe interactions rather than simple passive tolerance (30). Therefore, while the bacterioplankton would impact the gill microbiota, its composition would not completely mirror it (30) as host tissue will select for the most adapted strains (i.e., habitat selection/environmental filtering). Indeed, a difference (30) in composition can be seen in Fig. 2, with the gill microbiota mainly composed of *Betaproteobacteria, Flavobacteriia,* and *Gammaproteobacteria*, while the water microbiota was mainly composed of *Alphaproteobacteria, Bacilli,* and *Gammaproteobacteria.* The relative effects of the different factors on the gill microbiota of *S. rhombeus* are summarized in Fig. 7.

### Potential use of microbiota to detect cryptic species

The genotype effect on microbiota at the species level on skin and gut microbiota was demonstrated by Sylvain et al. (40) on piranha species (including black piranha), while Pratte et al. (17) observed the genotype effect on gill microbiota in different reef fish species. Based on this, we hypothesized that the putative cryptic species (group G) identified by Thomas et al. (38) would display a different microbiota signature from other groups. However, our findings did not support this hypothesis. Despite significant differences between group G and group FK in Fig. 3g and the presence of group-specific ASVs in group G, there were no distinctive signals observed in comparison to other genetic groups. Drawing clear conclusions on the potential use of microbiota biomarkers remains challenging. Indeed, in accordance with Sylvain et al. (3), our results indicate that fish gill microbiota is much more influenced by environmental factors than by host genotype, which most likely attenuated the influence of fish genotype. As previously mentioned, previous studies suggest that internal body parts are less impacted by the environment than skin or gill (15, 24, 55) and, as such, might be more suitable when employing microbiota as a tool for detecting cryptic species. This avenue warrants further exploration. Additionally, the cryptic species in our system is still hypothetical, and more tools are required to confirm its presence.

### Conclusion

Our study demonstrates that both environmental conditions and host genetics influence the gill microbiota activity of *Serrasalmus rhombeus*, influencing distinct ASVs. However, site-related factors, particularly bacterioplankton, were more strongly associated with variation in the gill microbial community. The diverse genetic clusters of *S. rhombeus* coexisting in sympatry and the ability of this ubiquitous species to thrive in contrasting environments render it an invaluable model for investigating the respective contribution of site-related factors and genetic factors on gill microbiota under natural conditions. Nonetheless, others’ site-related factors, such as parasites, pollutants, and food availability, were not addressed. Also, other host-specific factors, like individual health status, stress, and social behavior, need to be investigated and would provide a more comprehensive understanding. In addition, although bacterioplankton composition showed the strongest correlation with gill microbiota composition among the factors tested, much remains to be discovered regarding the interaction between host and microbiota, particularly in terms of how the host selects bacterioplankton. Further exploration is warranted to elucidate these dynamics fully.

## Data Availability

The genetic and environmental data are available in the Dryad Digital Repository (DOI: 10.5061/dryad.p8cz8wb00). Gill microbiome data of Serrasalmus rhombeus are available in the Sequence Read Archive (SRA) under BioProject IDs PRJNA901905 and PRJNA902720. Bacterioplankton microbiome data are available in the SRA under BioProject IDs PRJNA736442 and PRJNA736450.

## SUPPLEMENTAL MATERIAL

Figure S1 spectrum.03277-25-s0001.tif Relative abundance of the 100 most abundant ASV in *Serrasalmus rhombeus* gill microbiota and water communities (bacterioplankton).

Figure S2 spectrum.03277-25-s0002.tif Point distance from respective group centroids for B and K in site SOL-2, B and JD in site SOL-3, FK and G in site SOL-5, E and J in site NEG-3, and A and JIDL in site CUR.

Figure S3 spectrum.03277-25-s0003.tif Point distance from respective group centroids for BRA and NEG-3 containing genetic group B; SOL-6, SOL-1, and SOL-5 containing genetic group FK; and SOL-1 and SOL-2 containing genetic group K.

Figure S4 spectrum.03277-25-s0004.tif Predicted dissimilarity values by the global LMER model including environmental distance between site, genotype distance, and bacterioplankton dissimilarity.

Supplemental legends spectrum.03277-25-s0005.docx Descriptive legends for Fig. S1 to S4.

Supplemental tables spectrum.03277-25-s0006.docx Tables S1 to S6.

ASM does not own the copyrights to Supplemental Material that may be linked to, or accessed through, an article. The authors have granted ASM a non-exclusive, world-wide license to publish the Supplemental Material files. Please contact the corresponding author directly for reuse.
