# Supplementary figures and images for: Host genotype and environmental factors differentially shape the black piranha’s gill microbiota
Source: Microbiol Spectr. 2026 Apr 30;14(6):e03277-25. doi: 10.1128/spectrum.03277-25 (PMC13228033; doi:10.1128/spectrum.03277-25)

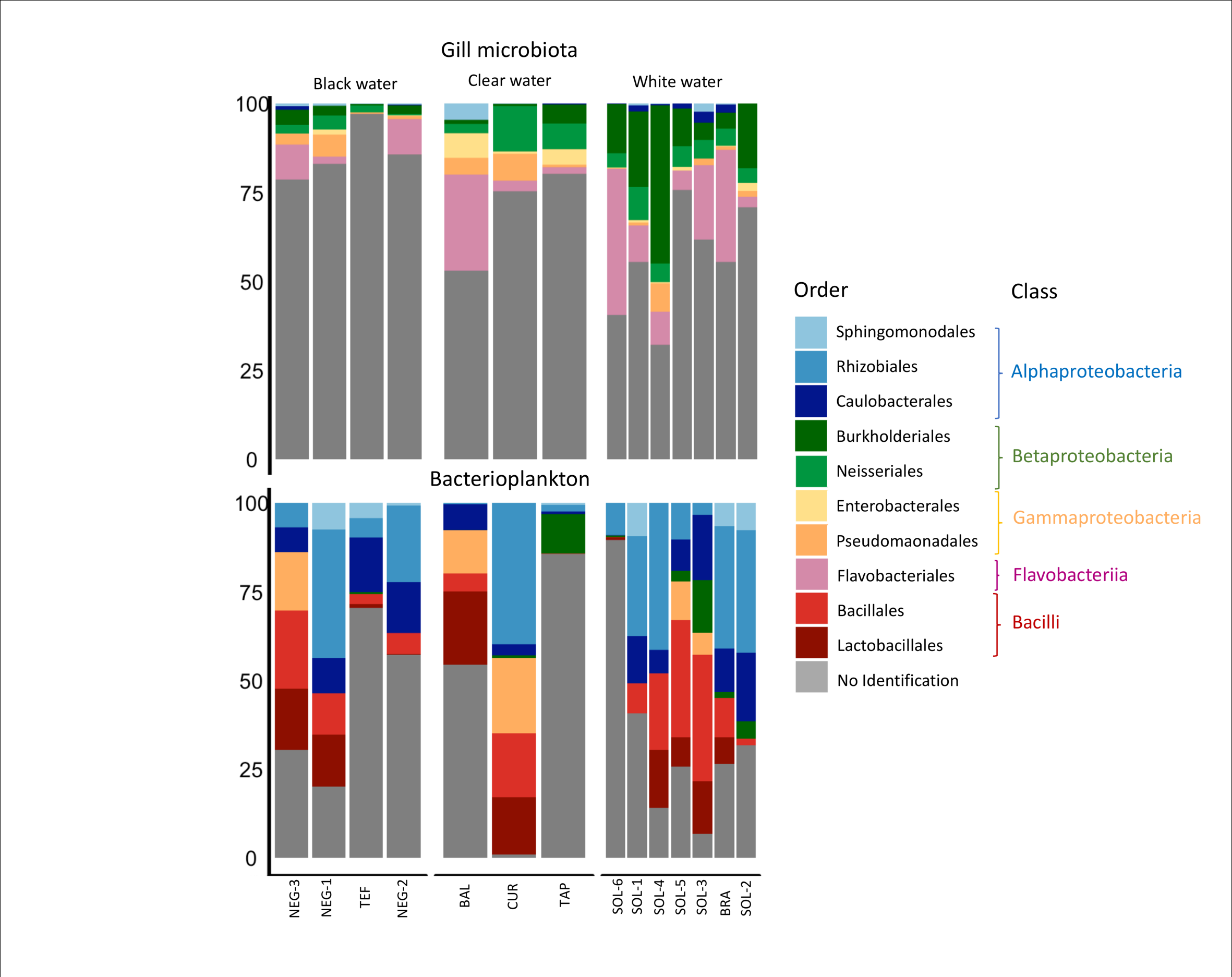

Supplement: Figure S1 — Relative abundance of the 100 most abundant ASV in Serrasalmus rhombeus gill microbiota and water communities (bacterioplankton). [file spectrum.03277-25-s0001.tif]

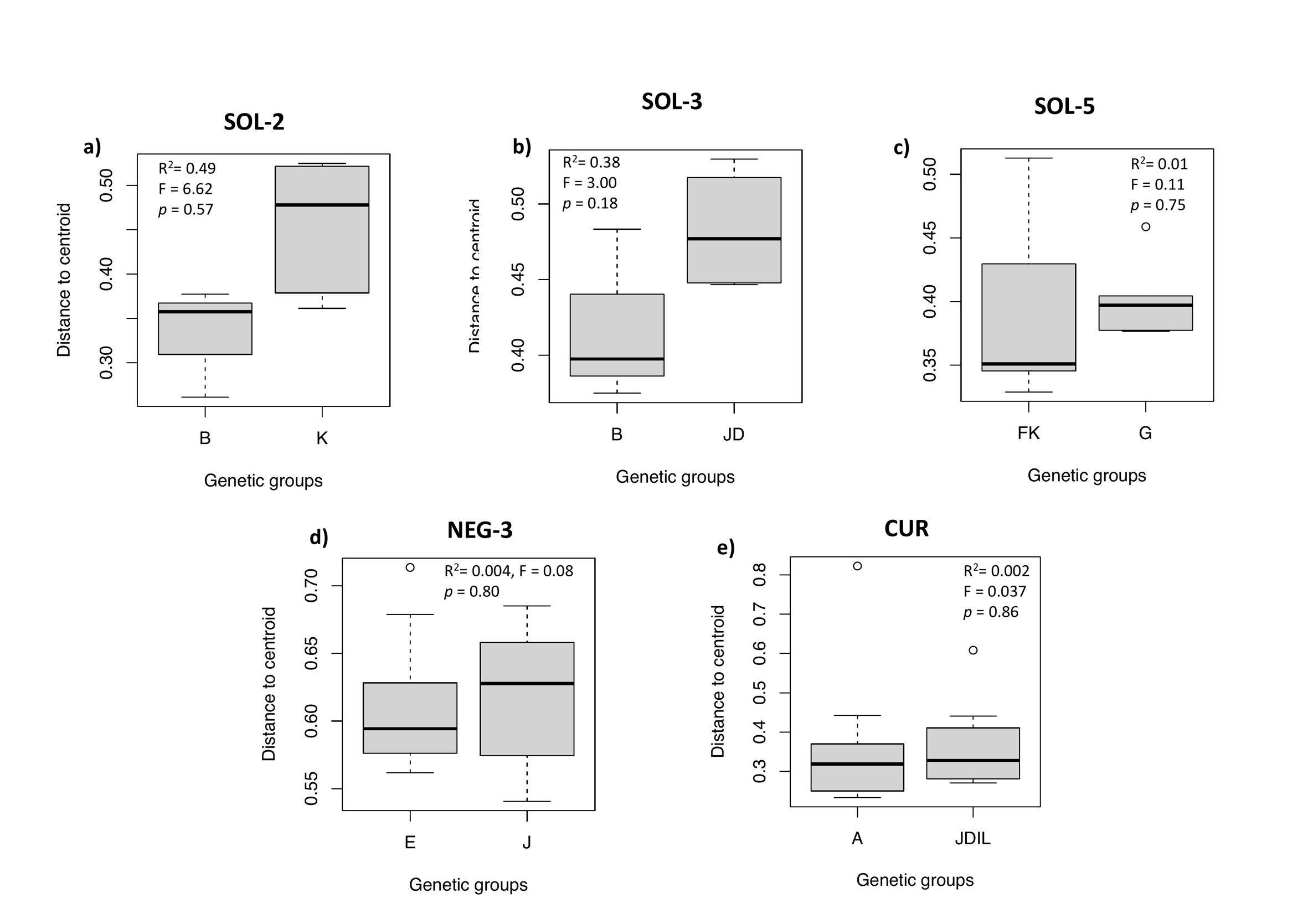

Supplement: Figure S2 — Point distance from respective group centroids for B and K in site SOL-2, B and JD in site SOL-3, FK and G in site SOL-5, E and J in site NEG-3, and A and JIDL in site CUR. [file spectrum.03277-25-s0002.tif]

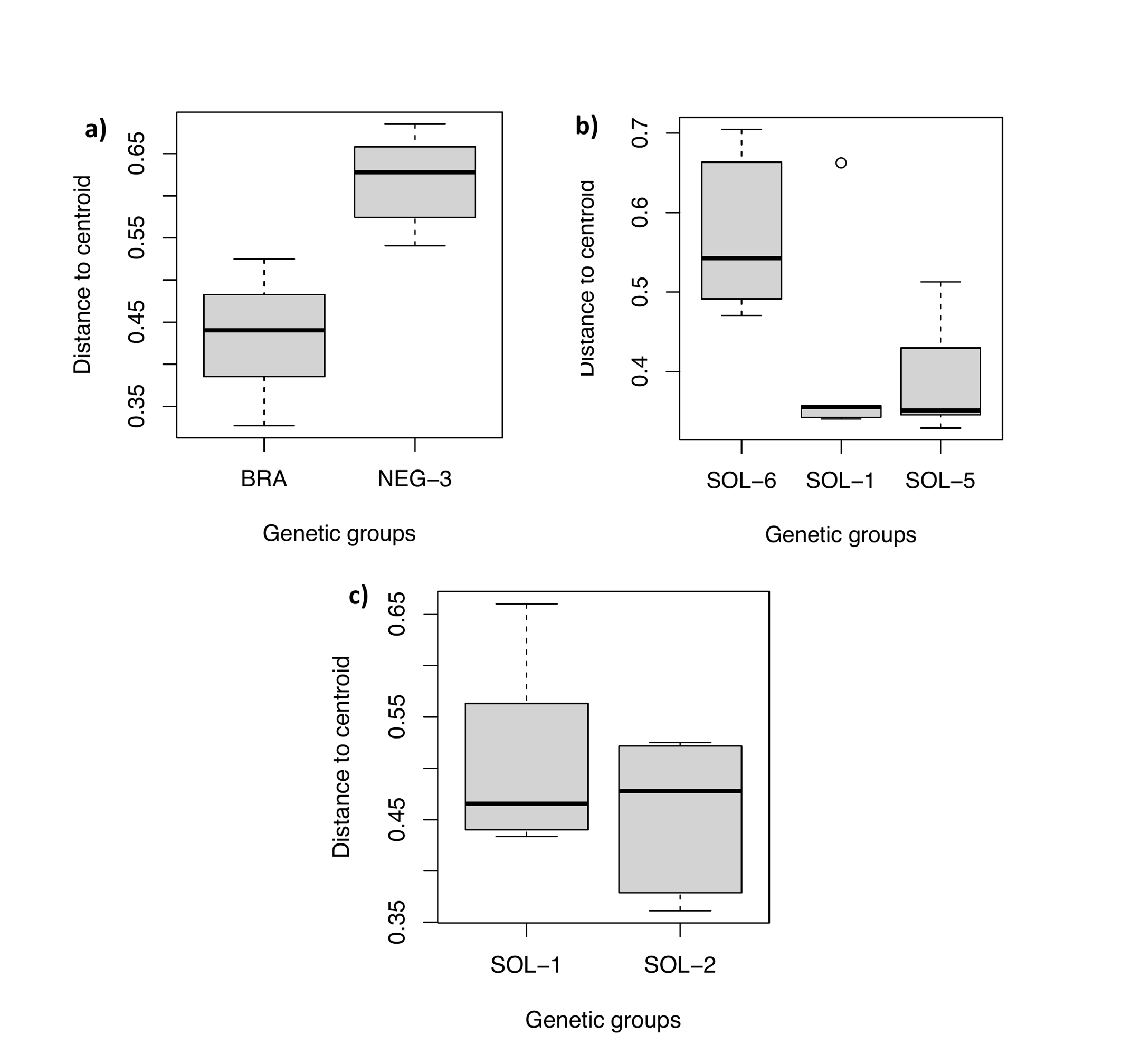

Supplement: Figure S3 — Point distance from respective group centroids for BRA and NEG-3 containing genetic group B; SOL-6, SOL-1, and SOL-5 containing genetic group FK; and SOL-1 and SOL-2 containing genetic group K. [file spectrum.03277-25-s0003.tif]

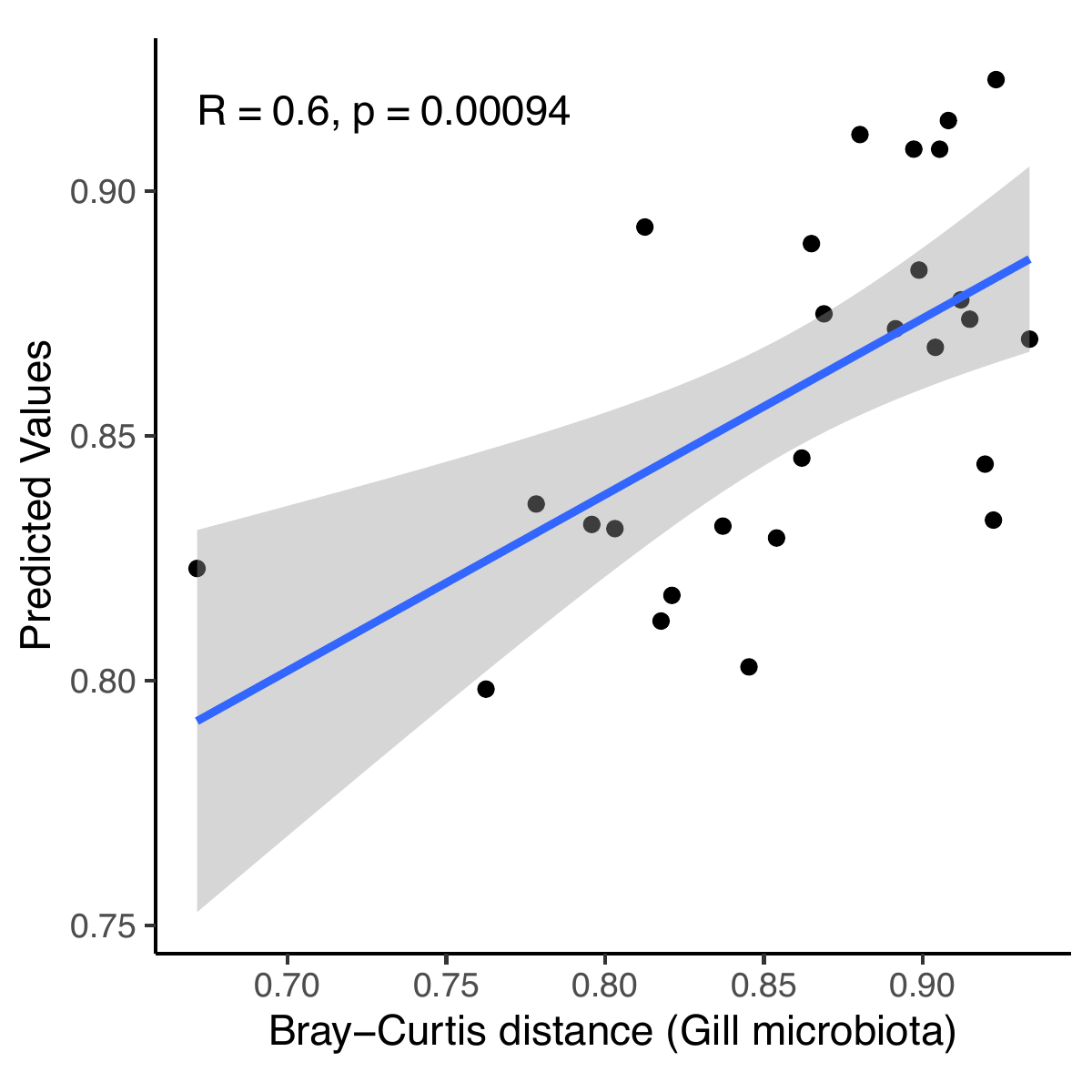

Supplement: Figure S4 — Predicted dissimilarity values by the global LMER model including environmental distance between site, genotype distance, and bacterioplankton dissimilarity. [file spectrum.03277-25-s0004.tif]
