# Supplementary material for: Host genotype and environmental factors differentially shape the black piranha’s gill microbiota
Source: Microbiol Spectr. 2026 Apr 30;14(6):e03277-25. doi: 10.1128/spectrum.03277-25 (PMC13228033; doi:10.1128/spectrum.03277-25)
Supplement: Supplemental legends — Descriptive legends for Fig. S1 to S4. [file spectrum.03277-25-s0005.docx]

**Supplementary figure 1** : Relative abundance of the 100 most abundant ASV in *Serrasalmus rhombeus* gill microbiota and water communities (bacterioplankton).

**Supplementary figure 2**: Point distance from respective group centroid for **a)** B and K in site SOL-2 (Betadisper test : R^2^= 0.49, F = 6.62, p = 0.06), **b)** B and JD in the site SOL-3 ( Betadisper test : R2 = 0.38, F = 3.00, p = 0.18) **c)** FK and G at the site SOL-5 (Betadisper test R2 = 0.01, F = 0.11, p = 0.75) d) E and J in the site NEG-3 (Betadisper test : R2 = 0.004, F= 0.08, p = 0.80) e) A and JIDL group in the site CUR ( Betadisper test : R2 = 0.002, F= 0.037, p = 0.86). Corresponding PERMANOVA results are found in fig.3.

**Supplementary figure 3**: Point distance from respective group centroid for a) BRA and NEG-3 containing the genetic group J (Betadisper test : R^2^ = 0.70, F = 55.76, p = 0.001***) b) SOL-6, SOL-1, SOL-5 containing the genetic group FK (Betadisper test : R^2^ = 0.46, F = 10.68, p = 0.002**) c) SOL-1 and SOL-2 sites containing geneic group K (Betadisper test : R^2^ = 0.12, F = 1.71, *p* = 0.194. Corresponding PERMANOVA results are found in fig.4.

**Supplementary figure 4**: Predicted dissimilarities values by the global LMER model including environmental distance between site, genotype distance and bacterioplankton dissimilarity.
