# Supplementary material for: Host genotype and environmental factors differentially shape the black piranha’s gill microbiota
Source: Microbiol Spectr. 2026 Apr 30;14(6):e03277-25. doi: 10.1128/spectrum.03277-25 (PMC13228033; doi:10.1128/spectrum.03277-25)
Supplement: Supplemental tables — Tables S1 to S6. [file spectrum.03277-25-s0006.docx]

**Supplementary material**

**Supplementary table 1:** Information on the sampling sites. The water type at each site was determined based on pH, dissolved organic carbon (DOC), optical characteristics of fluorescent dissolved organic matter (FDOM) and water conductivity.

| **Code** | **Site name** | **Water color** | **Watershed** | **Sampling period** | **n** |
| --- | --- | --- | --- | --- | --- |
| NEG-3 | Anavilhanas | Black | Rio Negro | 10/2018 | 20 |
| NEG-2 | Santo Alberto | Black | Rio Negro | 10/2019 | 20 |
| NEG-1 | Barcelos | Black | Rio Negro | 11/2018 | 20 |
| BRA | Rio Branco | White | Rio Branco | 10/2019 | 20 |
| SOL-6 | Lago Catalão | White | Rio Solimões | 10/2018 | 20 |
| SOL-5 | Lago Janauari | White | Rio Solimões | 10/2018 | 28 |
| SOL-4 | Lago Janauacá | White | Rio Solimões | 11/2018 | 16 |
| SOL-3 | Rio Manacapuru | White | Rio Solimões | 11/2018 | 7 |
| SOL-2 | Lago Téfé-Solimões | White | Rio Solimões | 11/2019 | 14 |
| SOL-1 | Lago dos Piratas | White | Rio Solimões | 11/2019 | 15 |
| TEF | Lago Tefé | Black | Tefé | 11/2019 | 17 |
| BAL | Réservoir Balbina | Clear | Rio Uatumã | 10/2018 | 17 |
| TAP | Rio Tapajós | Clear | Rio Tapajós | 10/2019 | 20 |
| CUR | Rio Curuá-Una | Clear | Rio Curuá-Una | 11/2018 | 20 |

**Supplementary table 2**: Measure of DOC quantity and FDOM optical characteristics of the water in which the *S.rhombeus* individuals were sampled

.

| **Site** | **DOC. Conc.** | **SAC340** | **SUVA254** | **Sbs254/365** | **% humic DOM** | **% Fulvic DOM** | **% Protein DOM** |
| --- | --- | --- | --- | --- | --- | --- | --- |
| **NEG-3** | 11.38 | 30.53 | 3.56 | 3.76 | 47.19 | 30.34 | 22.47 |
| **NEG-1** | 10.93 | 39.5 | 4.54 | 3.79 | 56.7 | 29.54 | 13.76 |
| **NEG-2** | 11.67 | 33.55 | 3.68 | 3.58 | 60.29 | 32.62 | 7.09 |
| **TEF** | 7.13 | 29.07 | 3.37 | 4 | 54.21 | 37.27 | 8.53 |
| **BAL** | 4.9 | 6.09 | 1.22 | 7.11 | 30.62 | 42.29 | 27.1 |
| **CUR** | 4.56 | 11.66 | 1.95 | 5.31 | 35.08 | 44.97 | 19.96 |
| **TAP** | 2.66 | 8.66 | 1.88 | 5 | 44.77 | 38.3 | 16.93 |
| **SOL-6** | 9.05 | 11.71 | 2.11 | 6.44 | 37.55 | 45.64 | 16.81 |
| **SOL-5** | 7.13 | 19.08 | 1.39 | 2.23 | 34.67 | 36.2 | 29.13 |
| **SOL-3** | 7.97 | 22.12 | 3.01 | 4.59 | 46.19 | 41.77 | 12.04 |
| **SOL-4** | 5.73 | 19.98 | 2.57 | 4.21 | 50.61 | 40.43 | 8.96 |
| **BRA** | 6.04 | 19.06 | 2.15 | 4.33 | 50.79 | 39.75 | 9.47 |
| **SOL-2** | 5.73 | 20.1 | 2.62 | 3.75 | 49.02 | 39.28 | 11.7 |
| **SOL-1** | 6.47 | 14.24 | 2.16 | 4.67 | 43.93 | 45.29 | 10.78 |

**Supplementary table 3 :** Concentrations of free ions and nutrients in the water where the *S.rhombeus* individuals were sampled.

| **Site** | **Na^+^** | **Mg^+2^** | **K^+^** | **Ca^+2^** | **Cl^-^** | **Nitrite** | **Nitrate** | **Silicate** |
| --- | --- | --- | --- | --- | --- | --- | --- | --- |
| **NEG-3** | 1.8 | 0.26 | 0.65 | 0.08 | 0.32 | 0.09 | 4.36 | 72.55 |
| **NEG-1** | 0.46 | 0.12 | 0.42 | 0.04 | 0.11 | 0.11 | 3.2 | 64.41 |
| **NEG-2** | 0.25 | 0.09 | 0.33 | 0.49 | 1.16 | 0.1 | 2.87 | 92.32 |
| **TEF** | 0.87 | 0.19 | 0.56 | 0.82 | 0.53 | 0.08 | 4.09 | 217.19 |
| **BAL** | 0.8 | 0.14 | 0.67 | 0.03 | 0.78 | 0.05 | 1.55 | 85.93 |
| **CUR** | 1.52 | 0.26 | 0.67 | 0.04 | 1.22 | 0.06 | 2.55 | 171.96 |
| **TAP** | 0.43 | 0.47 | 0.57 | 0.68 | 0.39 | 0.09 | 1.91 | 179.36 |
| **SOL-6** | 4.56 | 3.76 | 1.71 | 0.83 | 1.75 | 0.09 | 0.56 | 242.31 |
| **SOL-5** | 1.99 | 0.2 | 0.79 | 0.06 | 1.47 | 0.19 | 1.31 | 98.31 |
| **SOL-3** | 4.91 | 0.14 | 1.45 | 0.05 | 1.43 | 0.12 | 1.53 | 126.01 |
| **SOL-4** | 3.32 | 1 | 1.07 | 0.44 | 2.17 | 0.13 | 20.45 | 156.51 |
| **BRA** | 1.15 | 0.43 | 0.7 | 0.93 | 1.1 | 0.04 | 8.23 | 180.48 |
| **SOL-2** | 1.95 | 0.21 | 0.28 | 1.11 | 1.29 | 0.03 | 6.47 | 326.53 |
| **SOL-1** | 5.35 | 1.76 | 1.28 | 1.17 | 3.26 | 0.61 | 11.96 | 222.31 |

**Supplementary table 4 :**  Primary productivity characterization and measure of physicochemical parameters in the water where the *S.rhombeus* individuals were sampled.

| **Site** | **Chl a Conc.** | **Pheopigments Conc.** | **Chla/DOC** | **Temperature**  **(**°C) | **Cond.**  **(**uS) | **pH** | **% O_2_** |
| --- | --- | --- | --- | --- | --- | --- | --- |
| **NEG-3** | 0.05 | 0.38 | 0 | 30.7 | 13.2 | 4.24 | 53.2 |
| **NEG-1** | 0.35 | 2.43 | 0.03 | 31.6 | 13.1 | 3.71 | 92.12 |
| **NEG-2** | 0.73 | 0.33 | 0.06 | 30.6 | 10.6 | 4.16 | 58 |
| **TEF** | 1.82 | 1.73 | 0.26 | 30 | 10.6 | 4.98 | 61.5 |
| **BAL** | 0.83 | 0.78 | 0.17 | 33.2 | 16.8 | 5.05 | 103.2 |
| **CUR** | 1.25 | 2.38 | 0.28 | 31.2 | 19 | 6 | 79.1 |
| **TAP** | 2.15 | 1.03 | 0.81 | 30 | 14.1 | 6.36 | 80.2 |
| **SOL-6** | 7.14 | 6.6 | 0.79 | 32.9 | 174.8 | 5.7 | 44 |
| **SOL-5** | 4.62 | 17.31 | 0.65 | 32.9 | 22.4 | 4.38 | 60 |
| **SOL-3** | 2.78 | 10.54 | 0.35 | 32.6 | 24.3 | 5.31 | 72.8 |
| **SOL-4** | 1.35 | 1.88 | 0.24 | 29.3 | 88 | 6.75 | 82.6 |
| **BRA** | 6.21 | 2.89 | 1.03 | 31 | 22 | 6.25 | 88.7 |
| **SOL-2** | 4.41 | 3.2 | 0.77 | 30.3 | 19.7 | 6.05 | 68.6 |
| **SOL-1** | 9.05 | 4.69 | 1.4 | 31.9 | 127.6 | 7.15 | 31.9 |

**Supplementary table 5 :**  Concentration of dissolved metals (ug/L) in the water where the *S.rhombeus* individuals were sampled.

| **Site** | **Al** | **V** | **Cr** | **Mn** | **Fe** | **Co** | **Ni** | **Cu** | **Zn** | **As** | **Cd** | **Pb** |
| --- | --- | --- | --- | --- | --- | --- | --- | --- | --- | --- | --- | --- |
| **NEG-3** | 36.33 | 0.34 | 0.37 | 9.24 | 142.38 | 0.28 | 3.23 | 9.25 | 72.92 | 0.48 | 0.21 | 1.11 |
| **NEG-1** | 137.75 | 0.38 | 0.3 | 7.38 | 166.63 | 0.13 | 1.93 | 10.36 | 33.48 | 0.16 | 0.09 | 1.44 |
| **NEG-2** | 150 | 0.1 | 0.05 | 5.9 | 160 | 0.1 | 0.15 | 0.3 | 11 | 0.05 | 0.02 | 0.27 |
| **TEF** | 62 | 0.1 | 0.33 | 13 | 220 | 0.1 | 0.52 | 0.6 | 4.4 | 0.19 | 0.02 | 0.12 |
| **BAL** | 10.29 | 0.05 | 0.05 | 0.23 | 16.85 | 0.1 | 0.13 | 0.56 | 4.49 | 0.14 | 0.02 | 0.05 |
| **CUR** | 18.49 | 0.17 | 0.58 | 12.31 | 52.88 | 0.11 | 1.18 | 2.12 | 23.94 | 0.64 | 0.07 | 0.25 |
| **TAP** | 5 | 0.1 | 0.05 | 0.05 | 7 | 0.1 | 0.1 | 0.5 | 3.7 | 0.07 | 0.02 | 0.03 |
| **SOL-6** | 1.81 | 0.17 | 0.1 | 0.61 | 5.84 | 0.1 | 0.48 | 2.2 | 171.78 | 0.99 | 0.02 | 0.03 |
| **SOL-5** | 65.5 | 0.78 | 0.4 | 9.85 | 269.28 | 0.1 | 0.85 | 16.19 | 44.15 | 0.47 | 0.06 | 0.67 |
| **SOL-3** | 13.47 | 0.85 | 0.21 | 4.64 | 97.85 | 0.1 | 1.12 | 2.11 | 25.85 | 0.38 | 0.08 | 0.16 |
| **SOL-4** | 28.02 | 1.45 | 0.09 | 11.25 | 166.97 | 0.1 | 0.58 | 2.73 | 1.83 | 0.71 | 0.03 | 0.25 |
| **BRA** | 38 | 0.2 | 0.05 | 0.51 | 230 | 0.1 | 0.14 | 0.8 | 2.6 | 0.07 | 0.02 | 0.26 |
| **SOL-2** | 49 | 0.3 | 0.11 | 0.68 | 250 | 0.1 | 0.41 | 0.5 | 2.7 | 0.27 | 0.02 | 0.21 |
| **SOL-1** | 27 | 0.2 | 0.06 | 4.6 | 82 | 0.1 | 0.6 | 1.7 | 8.1 | 1.3 | 0.02 | 0.11 |

**Supplementary table 6 :** Results of the different LMER models tested.

| **Variables in the model tested** | **AIC Wt** |
| --- | --- |
| Bacterioplankton, physico chemical parameters, interaction between bacterioplankton and physicochemical parameters | 0.33 |
| Null model | 0.17 |
| Bacterioplankton,  physico chemical parameters, genotype and interaction between bacterioplankton and physicochemical parameters | 0.10 |
| Bacterioplankton | 0.09 |
| interaction between bacterioplankton and physicochemical parameters | 0.07 |
| Genotype | 0.6 |
| Eucledian distance | 0.06 |
| Bacterioplankton and genotype | 0.03 |
| Bacterioplankton and Euclidean distance | 0.03 |
| Bacterioplankton and interaction between bacterioplankton and physicochemical parameters | 0.03 |
| Genotype and Physico chemica parameters | 0.02 |
| Bacterioplankton,  physico chemical parameters  , genotype | 0.01 |
